# Multidimensional Mechanistic Spectrum of Long Non-coding RNAs in Heart Development and Disease

**DOI:** 10.3389/fcvm.2021.728746

**Published:** 2021-09-16

**Authors:** Lei Han, Lei Yang

**Affiliations:** Department of Pediatrics, Indiana University School of Medicine, Indianapolis, IN, United States

**Keywords:** long non-coding RNA, microRNA, heart development, heart disease, ceRNA, PRC2 complex

## Abstract

With the large-scale genome-wide sequencing, long non-coding RNAs (lncRNAs) have been found to compose of a large portion of the human transcriptome. Recent studies demonstrated the multidimensional functions of lncRNAs in heart development and disease. The subcellular localization of lncRNA is considered as a key factor that determines lncRNA function. Cytosolic lncRNAs mainly regulate mRNA stability, mRNA translation, miRNA processing and function, whereas nuclear lncRNAs epigenetically regulate chromatin remodeling, structure, and gene transcription. In this review, we summarize the molecular mechanisms of cytosolic and nuclear lncRNAs in heart development and disease separately, and emphasize the recent progress to dictate the crosstalk of cytosolic and nuclear lncRNAs in orchestrating the same biological process. Given the low evolutionary conservation of most lncRNAs, deeper understanding of human lncRNA will uncover a new layer of human regulatory mechanism underlying heart development and disease, and benefit the future clinical treatment for human heart disease.

## Introduction

The heart is a central organ of the circulatory system, which pumps blood and drives oxygen and nutrients throughout the whole body. According to American Heart Association, heart disease is one of the leading causes of death in the United States. Approximately 655,000 Americans die of heart disease each year (1). Although heart disease could be caused by various factors, the most direct and common reason has been recognized as genetic variations in coding genes. During the past decade, accumulated evidence demonstrates that non-coding RNAs (ncRNAs) are also highly relevant to cardiovascular diseases (2). Non-coding RNAs are transcripts without prominent protein coding potential, which include two major groups, short non-coding RNAs (sncRNAs) and long non-coding RNAs (lncRNAs) (3). SncRNAs include transfer RNAs (t-RNAs), ribosomal RNAs (r-RNAs), small nuclear RNAs (snRNAs), microRNAs (miRNAs), small interfering RNAs (siRNAs) and P-element-induced wimpy testis (PIWI) interacting RNAs (piRNAs). SncRNAs are broadly involved in transcriptional and translational regulations (3). LncRNAs are over 200 bp transcripts and lncRNA genes compose a large portion of the human genome. LncRNAs display multidimensional functions at various regulatory levels, such as histone modification, DNA methylation, gene transcription, post-transcription, translation, RNA and protein stability (4). Many lncRNAs have been reported to be involved in cardiovascular development and disease, although their underlying molecular mechanisms in pathological process remain elusive (5). Therefore, understanding the roles of lncRNAs in heart development and disease will reveal the molecular basis of cardiogenesis, and the molecular etiology of human cardiovascular diseases. For example, a conserved lncRNA *H19* represses cardiac hypertrophy by preventing and reversing experimental pressure-overload-induced heart failure, and Duchenne and Becker muscular dystrophy associated cardiomyopathy (6, 7). Hence, we summarize the current knowledge of characterized lncRNA mechanisms in heart development and disease (Table 1), and further discuss the clinical potential of lncRNA in heart disease therapy.

**Table 1:** Roles of lncRNAs in heart development and diseases.

	**Cellular location**	**Validated target(s)**	**Cardiac functions**	**References**
*HBL1*	Cytoplasm	*miR-1*	Cardiac development	(8, 9)
	Nucleus	JARID2 and EED		
*HOTAIR*	Cytoplasm	*miR-1*	Acute myocardial infarction	(10)
*LINCMD1*	Cytoplasm	*miR-133*; *miR-135*; *pre-miR-133b*	Myogenesis	(11)
*H19*	Cytoplasm	*let-7*; *miR-877-3p*; *miR-22-3p*; *miR-19a*; *miR-675-3p*; *miR-675-5p*; KSRP; Dystrophin.	Muscle differentiation and regeneration; MI-induced myocardial injury; Senescence; Diabetic cardiomyocyte; Muscular dystrophy	(6, 7, 12–20, 56)
	Nucleus	EED; EZH2; SUZ12		
*UCA1*	Cytoplasm	*miR-184*	Cardiac hypertrophy	(22)
*MIAT*	Cytoplasm	*miR-150*	Cardiac hypertrophy	(23)
*CHRF*	Cytoplasm	*miR-489*	Cardiac hypertrophy	(24)
*ROR*	Cytoplasm	*miR-133*	Cardiac hypertrophy	(25)
*Plscr4*	Cytoplasm	*miR-214*	Cardiac hypertrophy	(26)
*MALAT1*	Cytoplasm	*miR-220C*	Cardiomyocyte electrophysiology; cardiac remodeling and failure	(27–29)
	Nucleus	BRG1; HDAC9		
*CARL*	Cytoplasm	*miR-539*; *miR-296*	Cardiac apoptosis, replication, and regeneration	(30)
*CCRR*	Cytoplasm	CIP85	Cardiac conduction	(31)
*Meg3*	Cytoplasm	FUS	Cardiac apoptosis	(32)
*Bvht*	Nucleus	SUZ12	Cardiovascular lineage commitment	(33, 34)
*Fendrr*	Nucleus	PRC2; TrxG/MLL	Lateral plate or cardiac mesoderm differentiation	(35)
*PPP1R1B*	Nucleus	Ezh2	Myogenic differentiation	(36)
*Ahit*	Nucleus	SUZ12	Cardiac hypertrophy	(37)
*Chaer*	Nucleus	EZH2	Cardiac hypertrophy	(38)
*Uc.323*	Nucleus	EZH2	Cardiac hypertrophy	(39)
*Mhrt*	Nucleus	Brg1	Cardiac hypertrophy and failure	(41, 47)
*Linc1405*	Nucleus	Eomes	Cardiac differentiation	(42)
*CPR*	Nucleus	DNMT3A	Cardiac proliferation	(43)
*MDRL*	Cytoplasm	*miR-361*; *miR-484*	Cardiac apoptosis	(48)
	Nucleus	*Pre-miR-484*		

## Long Non-Coding RNA Functions in Heart Development and Disease

The establishment of *in vitro* cardiomyocyte (CM) differentiation from mouse and human pluripotent stem cells (hPSCs) allows modeling early events of cardiogenesis in dish. Furthermore, whole transcriptomic profiling and CRISPR/Cas-9 mediated approaches have paved the way toward discovering and functional assessment of crucial lncRNAs in early human cardiac development by using hPSCs (45). Currently, hundreds of lncRNAs have been identified in the human cardiac precursor cells (CPCs), such as cardiac mesoderm enhancer-associated non-coding RNA (*Carmen*), which promotes cardiac specification and differentiation of CPCs (46). A human-specific lncRNA, Heart Brake LncRNA 1 (*HBL1*), represses CM differentiation from human hPSCs via counteracting *miR-1* function (8). A mouse-specific lncRNA, *Braveheart* (*Bvht*), is required for the commitment of nascent mesoderm toward a cardiac fate (33). A heart field related lncRNA, *Linc1405*, controls cardiac mesoderm specification and cardiogenesis in mESC and *in vivo* (42). A lateral mesoderm-specific lncRNA *Fendrr* (FOXF1 Adjacent Non-Coding Developmental Regulatory RNA) plays an essential role in heart and body wall development *in vivo* (35). In addition to control early cardiac lineage specification, lncRNAs also play important roles in CM maturation and proliferation via various mechanisms, such as regulating the expression ratio of Myh6/Myh7 (47), sarcomere organization (43), cardiac myogenesis (36), metabolic maturation (44, 48) and cardiac conduction (27, 31, 49). LncRNA *Mhrt* (myosin heavy-chain-associated RNA transcripts) is required for maintaining the ratio of Myh6/Myh7 during mouse heart development and maturation, which is important for CM maturation (47). LncRNA *CPR* (cardiomyocyte proliferation regulator) induces hypertrophic responses of mature CMs, including increased sarcomere organization and CM surface area (43).

Evidence of the association between deregulation of lncRNAs and heart diseases has been reports for various cardiovascular disease models, such as cardiac hypotrophy (6), muscular dystrophy (7), coronary artery disease (CAD) (50–52), myocardial infarction (32, 53), diabetic cardiomyopathy (54), non-Ischemic cardiomyopathy (NICM) and heart failure (55). Murine and human lncRNA *H19* display an anti-hypotrophy function, and CM-restricted *H19* gene delivery can suppress the development of cardiac hypertrophy and later on heart failure (6). Recently, Zhang et al. found that *H19* inhibits dystrophin degradation, preserves skeletal and cardiac muscle histology, and improves cardiomyocyte strength and heart function in muscular dystrophy cells and murine model (7). *H19* also suppresses apoptosis and autophagy of CMs under diabetic condition (12, 56). In myocardial infarction, lncRNA *Meg3* is upregulated in infarcted mouse heart and promotes CM death (32). Although a large number of lncRNAs have been found to be associated with heart development and disease (Table 1), the mechanisms of most lncRNAs remain elusive. Particularly, the deeper understating of lncRNA mechanisms will shed light on the clinical potential of lncRNAs, with the findings of novel therapeutic targets or druggable lncRNAs. Interestingly, many lncRNAs show restricted expression patterns in the cytoplasm or nucleus although some lncRNAs express in both, suggesting the differential functions executed by lncRNAs in different subcellular localizations, which are summarized in the following sections.

## Mechanisms of Long Non-Coding RNAs in Cytoplasm

The subcellular localization is considered as a key factor determining lncRNA function (57, 58). Although the nucleus is the location for RNA biogenesis and processing, many mature lncRNAs are transported into cytoplasm, showing high cytosolic expressing levels (59). In the cytoplasm, lncRNA-mediated mechanisms have been found to mainly regulate mRNA stability, translation of mRNA, and microRNA (miRNA) related functions (60).

### Long Non-coding RNA Counteracts microRNA

**Figure 1:**
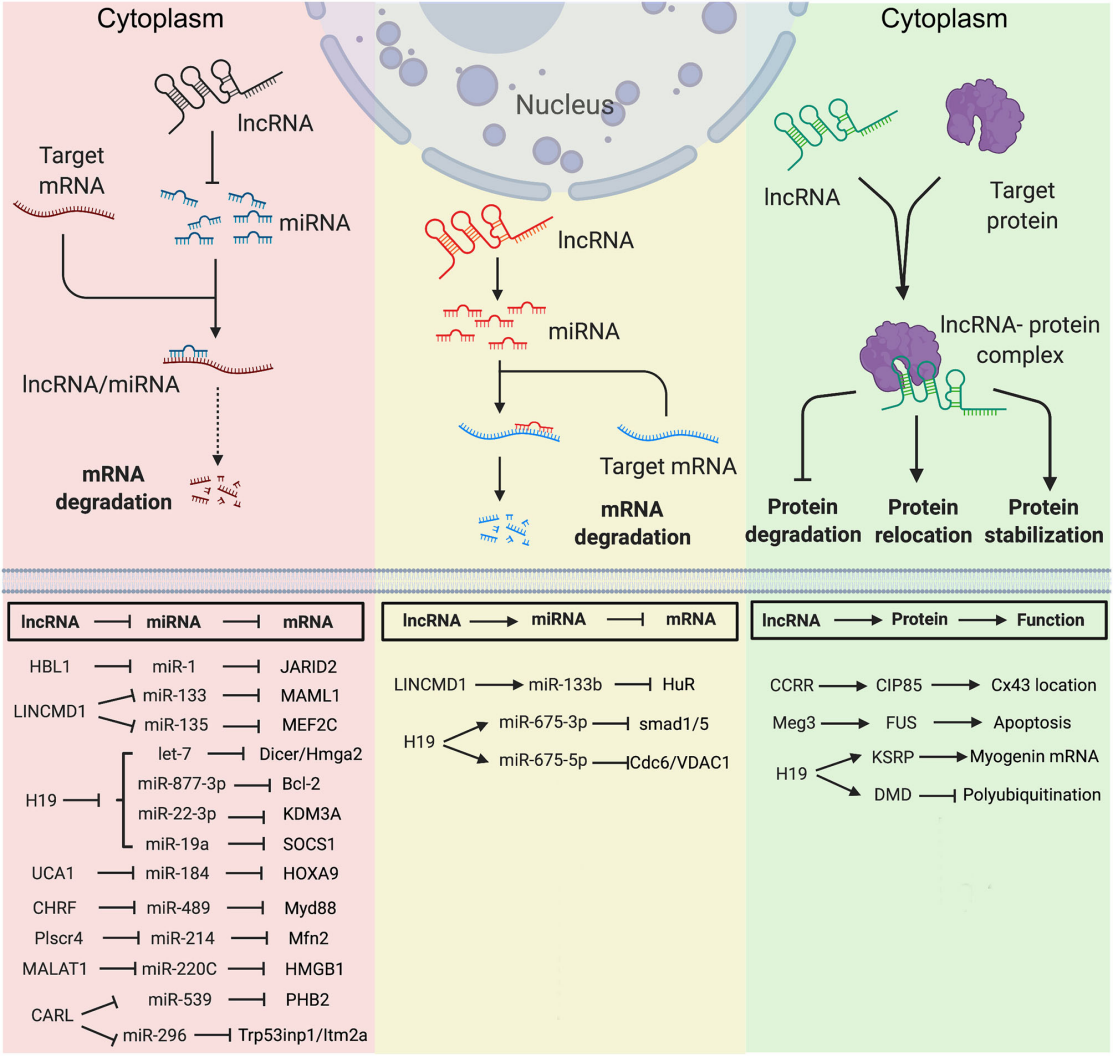
Mechanisms of Long non-coding RNAs in cytoplasm in heart development and diseases. **(Left)** lncRNA functions as miRNA sponge. **(Middle)** lncRNA functions as miRNA precursor. **(Right)** lncRNA functions as protein scaffold. Created with BioRender.com.

Since the first discovery of competing endogenous RNA (ceRNA), hundreds of lncRNAs have been found to function as miRNA sponge to counteract endogenous miRNAs. The ceRNAs can modulate miRNA activity through sequestration, thereby increasing the expression of miRNA target genes (61). During heart development, several lncRNAs have been identified to counteract miRNAs and regulate expressions of genes essential for stem cells pluripotency or lineage specification. Using hPSCs, *HBL1* was identified as a modulator to fine-tune human CM development via sponging *miR-1* (8). *HBL1* is a human-specific lncRNA highly expressed in hPSCs and gradually diminishes during CM differentiation. Loss of *HBL1* increases CM differentiation from hPSCs. *HBL1* expresses in both nucleus and cytoplasm of undifferentiated hPSCs. In the cytoplasm, *HBL1* binds with *miR-1* to fine-tune its activity and further regulate cardiogenic gene expressions (Figure 1). Additionally, lncRNA *HOTAIR* (HOX antisense intergenic RNA), which was initially described as a regulator of cancer progression, also displays a cardioprotective role in acute myocardial infarction, which is partially through the interaction and negative regulation of *miR-1* (10).

*LINCMD1* (Long Intergenic Non-protein Coding RNA, Muscle Differentiation 1) is a muscle-specific ceRNA, which is required for muscle differentiation and plays an important role in myogenesis. *LINCMD1* acts as ceRNAs for two muscle-specific microRNAs, *miR-133* and *miR-135*, which target the MAML1 (expression of mastermind-like-1) and MEF2C (myocyte-specific enhancer factor 2C) genes, respectively (Figure 1). MAML1 and MEF2C are transcriptional coactivators which positively regulate muscle-specific gene expression. Depletion of *LINCMD1* represses the expression of MAML1 and MEF2C, whereas overexpression of *LINCMD1* increases MAML1 and MEF2C expression levels and promotes muscle differentiation (11).

It was reported that approximately 378,295 ceRNA interactions appeared in the cardiovascular disease-related ceRNA interactions (62). *H19* is a lncRNA with high expression level in embryos (63, 64). *H19* is conserved in both human and mouse and has no coding potential. *H19* is required for muscle differentiation and regeneration via acting as a natural molecular sponge for the *let-7* family of miRNAs (13). Depletion of *H19* causes precocious muscle differentiation, which can be repressed by *let-7* overexpression (Figure 1) (14). In H_2_O_2_-treated CMs and mouse ischemia-reperfusion (I/R) hearts, *H19* functions as a ceRNA for *miR-877-3p*, which targets Bcl-2 to further regulate mitochondria-mediated apoptosis in myocardial I/RI (Figure 1) (15). Additionally, Zhang et al. reported that *H19* functions as a ceRNA of *miR-22-3p*, which directly targets KDM3A gene to ameliorate MI-induced myocardial injury (Figure 1) (16). *H19* is also a pro-senescence lncRNA in CMs by counteracting *miR-19a* to upregulate SOCS1 expression and further activate the p53/p21 pathway to promote CM senescence (Figure 1) (17).

Many lncRNAs have been reported to play a ceRNA role in hypertrophic cardiomyopathy. LncRNA *UCA1* regulates cardiac hypertrophy via the *UCA1*/*miR-184*/HOXA9 axis (Figure 1) (22). *MIAT* promotes cardiac hypertrophy through targeting *miR-150* (23). LncRNA *CHRF* (cardiac hypertrophy related factor) regulates cardiac hypertrophy via the *CHRF*/*miR-489*/Myd88 axis (24). LncRNA *ROR* mediates cardiac remodeling and promotes cardiac hypertrophy via interacting with *miR-133* (25). *Plscr4* negatively regulates cardiac hypertrophy *in vivo* and *in vitro* via the *miR-214*/Mfn2 axis (26). *MALAT1* (metastasis-associated lung adenocarcinoma transcript 1) reduces transient outward potassium current of CMs by targeting *miR-220C* and its downstream target gene HMGB1 (Figure 1) (27). *CARL* (cardiac apoptosis-related lncRNA) significantly increases in CMs since the neonatal stage of mouse (44). *CARL* can negatively regulate mitochondrial fission and apoptosis through the *miR-539*/PHB2 axis (44). It can also directly target *miR-296* and its downstream genes Trp53inp1 and Itm2a, further regulating CM replication and cardiac regeneration after injury (Figure 1) (30).

To date, hundreds of publications have reported the ceRNA role of lncRNAs under normal and diseased conditions. Given the cascading effects exerted by the gene networks comprising ceRNA-miRNA-coding genes, lncRNA and its downstream gene networks are potential new targets for cardiovascular disease therapy.

### Long Non-coding RNA Forms miRNA Precursor

LncRNAs can be transcribed as miRNA precursors, which produce mature miRNAs via further processing. Therefore, lncRNAs could indirectly regulate the expression of miRNA downstream target genes. For example, transcript of *LINCMD1* hosts a *pre-miR-133b* transcript. The RNA-binding protein HuR is a component of *LINCMD* regulatory circuitry to regulate muscle differentiation (65). During the early stage of muscle differentiation, HuR binds to *LINCMD1* and promotes *miR-133* biogenesis from the *LINCMD1* transcript. HuR/*LINCMD1* complex is then targeted by *miR-133* in the cytoplasm (Figure 1). Thus, the ceRNA function of *LINCMD1* reinforces HuR expression via counteracting *miR-133* in a positive feedforward loop (65). In this case, *LINCMD1* plays dual roles in fine-tuning the dynamic of muscle differentiation and regeneration.

Interestingly, the exon 1 of *H19* hosts transcripts of *miR-675-3p* and *miR-675-5p*. *MiR-675-3p* regulates the bone morphogenetic protein (BMP) signaling pathway by directly targeting Smad1 and Smad5 mRNAs (Figure 1) (18). *MiR-675-5p* could target DNA replication initiation factor Cdc6 mRNA (18). Therefore, *H19* exhibits a pro-differentiation function in primary myoblasts and regenerating skeletal muscles (19). In the rat model of diabetic cardiomyopathy, overexpression of *H19* can attenuate apoptosis of diabetic CMs and improve left ventricular function, whereas knockdown of *H19* shows opposite functions. Mechanistically, *H19* expression is significant downregulated in the hearts of rats with diabetic cardiomyopathy, which leads to a reduced level of *miR-675* and an increased level of *miR-675* target-gene VDAC1. Enhanced VDAC1 can induce apoptosis of CMs when exposed to high glucose (12).

### Long Non-coding RNA Functions as Protein Scaffold

In cytoplasm, lncRNA can regulate protein location and stability by directly binding with target protein(s). As an anti-arrhythmic lncRNA, *CCRR* (cardiac conduction regulatory RNA) is downregulated in both mouse and human heart failure (31). *CCRR* knockdown induces arrhythmias, and its overexpression improves cardiac conduction. *CCRR* is also required for maintaining the proper distribution of connexin43 (CX43) in the intercalated discs (Figure 1). Mechanically, *CCRR* directly binds with CX43-interacting protein CIP85 and prevents CX43 from backward trafficking and subsequent degradation in the cytoplasm of CMs (31).

*Meg3* is upregulated in infarcted mouse hearts and human failing hearts. *Meg3* expression is directly regulated by p53 under hypoxic condition. It has been reported that *Meg3* has a pro-apoptotic function in rodent CMs (32). *Meg3* shRNA delivered by the adeno-associated virus serotype 9 (AAV9) can significantly improve cardiac function. *Meg3* functions as protein scaffold to direct bind with RNA-binding protein FUS and regulates apoptotic signaling pathway (Figure 1) (32).

Except for the functions mentioned above, *H19* also interacts with proteins in the cytoplasm. In the undifferentiated multipotent mesenchymal C2C12 cells, *H19* interacts with a multifunctional RNA binding protein KSRP (K homology-type splicing regulatory protein) (20). To maintain the undifferentiated state of C2C12 cells, cytoplasmic *H19* post-transcriptionally modulates gene expression via acting as a protein scaffold of KSRP and promotes its interaction with RNA exosome, which further enhances the KSRP-promoted mRNA decay of myogenic genes (20). Recently, in muscular dystrophy (MD) patients, *H19* was found to directly interact with dystrophin and inhibit E3-ligase-dependent polyubiquitination at Lys3584 for protein degradation. Non-silent mutation (C3340Y) of dystrophin results in defective interaction between dystrophin and *H19*, which causes ubiquitination and degradation of dystrophin (Figure 1) (7). In both *Dmd* mouse model and human iPSC-derived skeletal muscle cells from patients with Becker MD, simultaneous administration of *H19* RNA mimic and nifenazone, an analgesic for rheumatic conditions, could effectively inhibit dystrophin degradation, preserve skeletal and cardiac muscle histology, and improve cardiac strength and heart function. This suggests a protective role of *H19* in both Becker and Duchenne muscular dystrophy, providing a potential RNA therapy for MD patients (7).

## Long Non-Coding RNA Functions in Nucleus

Compared to cytoplasm, RNAs are processed in nucleus where many lncRNAs reside and execute functions. Nuclear lncRNAs play a variety of crucial roles with complex molecular mechanisms, including regulating chromatin organization, transcription, and different nuclear condensates (66).

### Long Non-coding RNA Interacts With the Polycomb-Repressive Complex 2 (PRC2)

**Figure 2:**
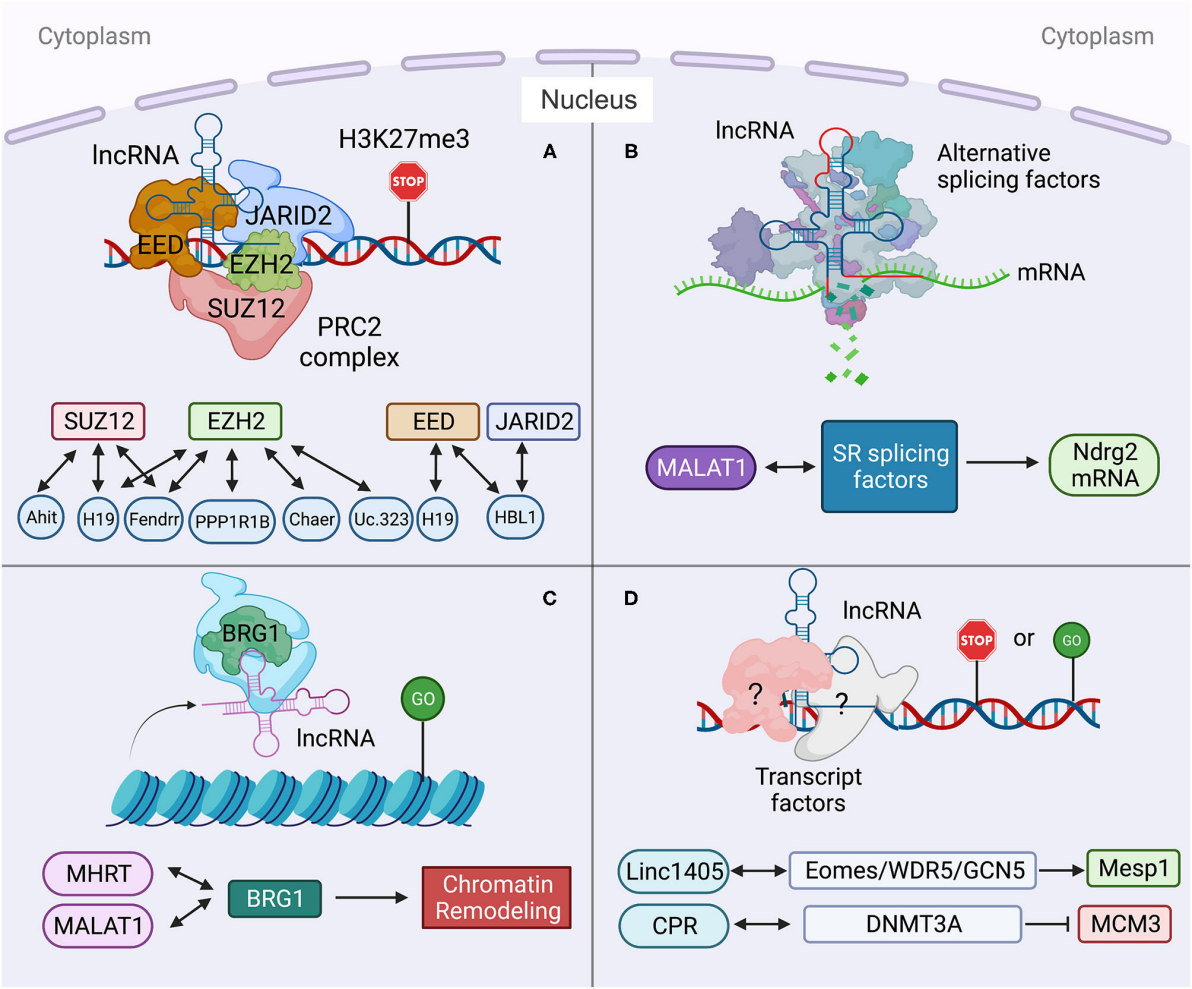
Long non-coding RNA mechanisms in nucleus in heart development and diseases. **(A)** lncRNA targets to PCR2 complex and regulates its downstream gene transcription. **(B)** lncRNA regulates pre-mRNA alternative splicing. **(C)** lncRNA regulates chromatin remodeling. **(D)** lncRNA interacts with transcription factors to regulate gene transcription. Created with BioRender.com.

Multiple nuclear lncRNAs have been found to regulate lineage differentiation by interacting with PRC2. Histone-modifying complex PRC2 plays a pivotal role in determining the epigenetic state of genes controlling pluripotency, lineage commitment, and cell differentiation (67). A heart-associated lncRNA, *Bvht* is required for the commitment of nascent mesoderm to a cardiac fate from mouse ESCs (33). In the nucleus, *Bvht* can activate the core cardiovascular gene network by interacting with SUZ12, a component of PRC2, during CM differentiation (Figure 2). In *Bvht*-depleted cells, SUZ12 and PRC2 associated chromatin modification H3K27me3 are deposited at promoters of cardiogenic genes, such as Mesp1, which is a master regulator of cardiovascular fate commitment (33). Additionally, deletion of a 5′ asymmetric G-rich internal loop (AGIL) in *Bvht* can dramatically impair CM differentiation (34). Through AGIL, *Bvht* can interact with a cellular nucleic acid binding protein CNBP (ZNF9), which is known as a zinc-finger protein to bind with single-stranded G-rich sequences. Together, in the nucleus, *Bvht* controls cardiovascular lineage commitment by interacting with SUZ12/PRC2 and CNBP through defined RNA motifs (33, 34).

In mouse, a lateral mesoderm-specific lncRNA *Fendrr* is essential for heart development (35). During mouse embryo development, *Fendrr* binds with both PRC2 via the EZH2 subunit and TrxG/MLL complexes and acts as modulators of PRC2 or TrxG/MLL activity (Figure 2) (35). *Fendrr* deficient embryos show upregulation of several transcription factors controlling lateral plate or cardiac mesoderm differentiation, accompanied with a drastic reduction of PRC2 occupancy and decreased H3K27 trimethylation and/or increased H3K4 trimethylation at those gene promoters. So, similar to *Bvht, Fendrr* plays an essential role in controlling cardiac lineage fate commitment via PRC2 (35).

The interaction between lncRNA and PRC2 complex is conserved in human and mouse. LncRNA *PPP1R1B* was found to bind with EZH2, a key PRC2 subunit (Figure 2) (36, 68). Silencing of *PPP1R1B* compromises myotube development in both mouse C2C12 and human skeletal myoblasts (36). In hiPSCs-CMs, *PPP1R1B* deficient also impairs myogenic differentiation (36). *PPP1R1B* regulates the expression of myogenic transcription factors, such as MyoD, Myogenin, and Tbx5, by interacting with PRC2 at the chromatin interface. *PPP1R1B* interacts with PRC2 to suppress H3K27me3 histone modification on the MyoD1 and Myogenin promoters. In the nucleus, *PPP1R1B* modulates PRC2 occupancy on promoters of essential myogenic genes to regulate myogenic differentiation during heart and skeletal muscle development (36).

Our recent study found that nuclear *HBL1* interacts with two PRC2 subunits, JARID2 and EED in human pluripotent stem cells (Figure 2) (9). During human cardiogenesis, loss of *HBL1* disrupts genome-wide PRC2 occupancy, reduces H3K27me3 chromatin modification on essential cardiogenic genes, and therefore enhances cardiogenic gene transcription in undifferentiated hPSCs and later-on differentiation. At the pluripotency stage, deletions of *HBL1* and JARID2 both reduce PRC2 occupancy on 62 overlapped cardiogenic genes. Therefore, *HBL1* precisely controls cardiogenic gene transcription via modulating PRC2 occupancy.

*H19* plays important functions in both cytoplasm and nucleus. In diabetic cardiomyopathy, cytosolic *H19* forms *miR-675-3p* and *miR-675-5p* and attenuates apoptosis of CMs (12). Under the same pathological condition, Zhuo et al. reported that *H19* directly binds with EZH2, a subunit of PRC2, in CM nucleus to affect the anti-autophagy function (56). Loss of H19 was found to reduce EZH2 and H3K27me3 occupancy on the promoter of DIRAS3, which regulates the formation of autophagosome initiation complex (Figure 2) (21), and causes DIRAS3 downregulation. Consistent with its cytosolic function (12), overexpression of *H19* can inhibit cell death of CMs caused by high glucose via this nuclear mechanism. Recently, Viereck et al. reported the interaction between *H19* and PRC2 complex subunits EED, EZH2 and SUZ12 in the nuclear lysate of HL-1 CMs (Figure 2) (6). In pressure overload-induced left ventricular hypertrophy mice, *H19* ablation aggravates cardiac hypertrophy compared to wild-type mice. Taken together, *H19* physically interacts with PRC2 to suppress H3K27me3 modification at the Tescalcin locus, which is an anti-hypertrophic gene, to promote Tescalcin expression and in turn repress the NFAT signaling pathway (6).

Many other lncRNAs also have been found to interact with PRC2 complex under heart disease conditions. *Ahit* suppresses cardiac hypertrophy through binding with SUZ12 to regulate PRC2 occupancy on the MEF2A (myocyte enhancer factor 2A) promoter (Figure 2) (37). *Chaer* is required for the development of cardiac hypertrophy through direct binding with PRC2 subunit EZH2 to further regulate expressions of Anf, Myh7 and Acta1 genes (Figure 2) (38). *Uc.323* protects CMs against cardiac hypertrophy by binding with EZH2 to regulate CPT1b gene expression (Figure 2) (39).

Taken together, lncRNAs play important roles in cardiac development and diseases by interacting with PRC2 complex to affect PRC2-related epigenetic modifications.

### Long Non-coding RNA Regulates Alternative Splicing of Pre-mRNA

Alternative splicing (AS) of pre-mRNA enhances diversities of transcriptome and proteomic of the genome in higher eukaryotes (69). During tissue- or cell-type specification, the serine/arginine (SR) splicing factors regulate AS in a concentration or phosphorylation dependent manner (70, 71). During human cardiovascular differentiation, stage-specific RNA alternative splicing and lineage-enriched lncRNAs were identified by whole RNA-seq (45). As a long nuclear-retained regulatory RNA (nrRNA), *MALAT1* interacts with SR splicing factors in the nuclear speckle domains (Figure 2) (28). *MALAT1* regulates mRNA alternative splicing by modulating the levels of phosphorylated SR proteins (28). During pressure overload-induced cardiac remodeling and failure, *Malat1* was found to be an alternative splicing regulator of Ndrg2, which shows skipped exon 3 in hypertrophic mouse hearts (72, 73).

### Long Non-coding RNA and Chromatin Remodeling

In addition to interactions with splicing factors and epigenetic factors, lncRNAs have also been shown to interact with chromatin remodeling complexes (74). *Mhrt* (myosin heavy-chain-associated RNA transcripts) is a cardiac-specific lncRNA located in the murine myosin heavy chain 7 locus and is suppressed by the BRG1-HDAC-PARP chromatin repressor complex in cardiomyopathy (75). Overexpression of *Mhrt* protects mouse heart from hypertrophy and failure (47). *Mhrt* directly binds with BRG1, which is a chromatin-remodeling factor and the ATPase subunit of the SWI/SNF complex (Figure 2) (40), to remove SWI/SNF from its occupied genomic regions on target genes, thus regulating chromatin remodeling and gene transcription. *Mhrt* binds with the helicase domain of BRG1, which is crucial for tethering BRG1to its targets. In turn, BRG1 represses *Mhrt* in stress-induced cardiac hypertrophy and failure (41). This *MHRT*-BRG1 feedback circuit is also conserved in the human heart (47). *MALAT1* can also form RNA-protein complex with chromatin-remodeling enzyme BRG1 and histone deacetylase HDAC9 in vascular smooth muscle cells (Figure 2). This HDAC9-*MALAT1*-BRG1 complex represses expression of contractile protein genes in association with gain of H3K27me3 histone modification (29).

### Long Non-coding RNA Interacts With Transcription Factors

Besides chromatin-remodeling factors and epigenetic factors, transcription factors have also been found to interact with lncRNAs in heart development and disease. *Linc1405* is highly expressed in heart during mouse embryo development and critical for proper cardiac differentiation (42). *Linc1405* interdependently interacts with Eomes, which physically mediates Eomes/WDR5/GCN5 complex binding at the enhancer region of Mesp1 gene to activate its expression (Figure 2) (42). Mesp1 is one of the earliest key regulators of cardiac lineage specification (76). Disruption of Mesp1 in mice results in embryonic lethality due to a cardiac mesoderm deficiency (77). Therefore, *linc1405* guides Eomes/WDR5/GCN5 complex to directly target Mesp1 and affect expression of Mesp1 downstream genes to control cardiac differentiation (42).

Recently, lncRNA *CPR* (cardiomyocyte proliferation regulator) was found to play an important role in the regulation of CM proliferation (43). Deletion of *CPR* in CMs increases CM proliferation, reduces scar formation, and improves heart function after myocardial injury. Mechanically, *CPR* represses CM proliferation by suppressing the transcription of MCM3, which regulates initiation of eukaryotic genome replication and cell cycle (78) by direct binding with DNMT3A. Further, DNMT3A promotes CpG methylation of MCM3 promoter and represses transcription of MCM3 (Figure 2) (43).

## Crosstalk of Cytosolic and Nuclear Portions of the Same Long Non-coding RNA

**Figure 3:**
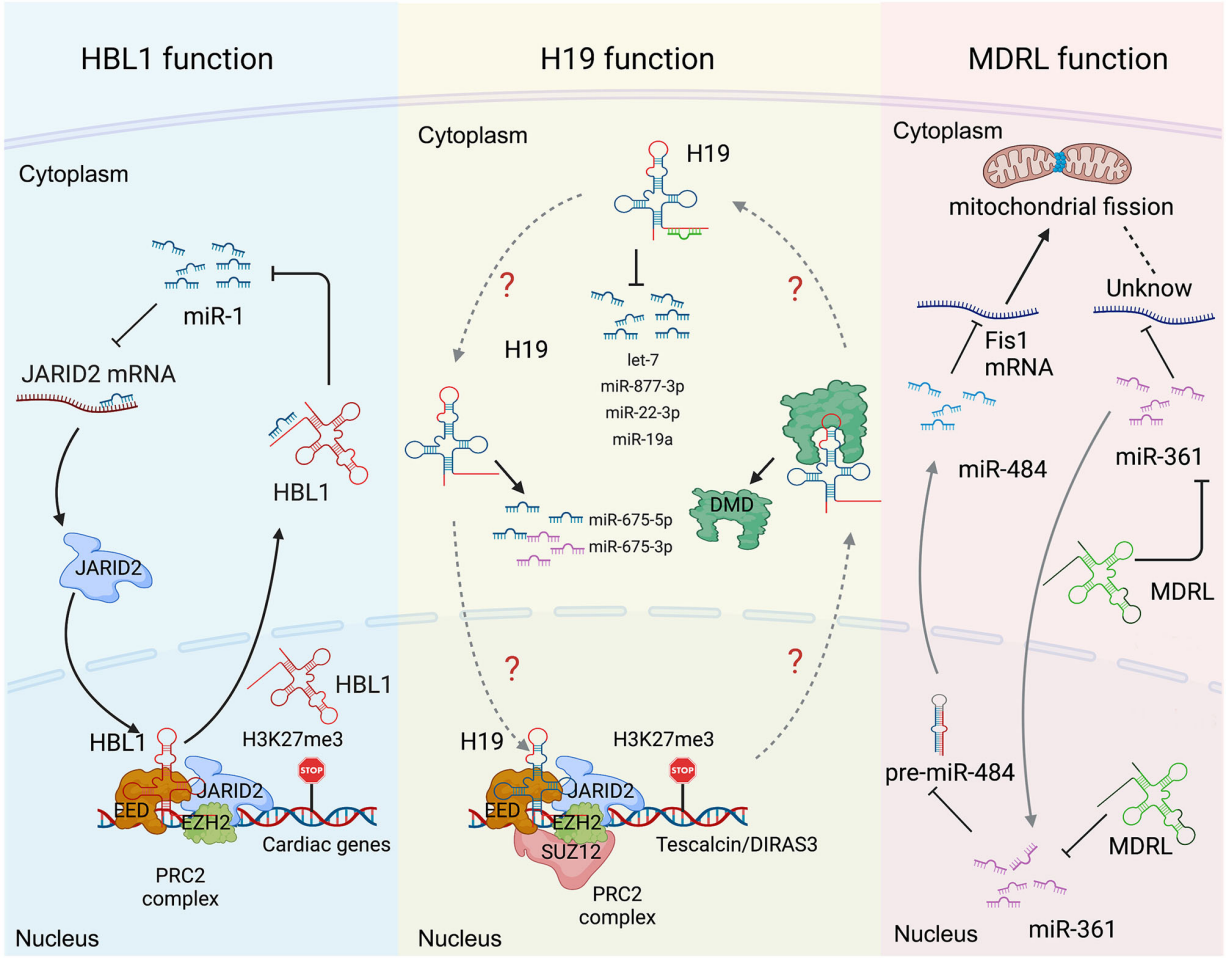
Long non-coding RNA mechanisms cohere with cytoplasm and nucleus in heart development and diseases. **(Left)** the functions of *HBL1* in both cytoplasm and nucleus. **(Middle)** *H19* has multiple functions in both cytoplasm and nucleus. **(Right)** the functions of *MDRL* in both cytoplasm and nucleus. Created with BioRender.com.

Many lncRNAs, such as *HBL1* (8, 9), *H19* (6, 7, 13, 14, 19, 20), *MDRL* (48) and *LncMyoD* (79), express in both cytoplasm and nucleus to display different functional mechanisms. However, how the cytosolic and nuclear mechanisms mediated by the same lncRNA could crosstalk with each other has been rarely studied. Recently, we reported the function of nuclear *HBL1* in human cardiogenesis (9), following our previous characterization of cytosolic *HBL1* role during human CM differentiating (8). We also defined the mechanism by which cytosolic and nuclear *HBL1* crosstalk to control cardiogenic gene transcription (9). *HBL1* functions as a *miR-1* sponge in cytoplasm and governs *PRC2* occupancy on cardiogenic genes in nucleus (Figure 3). In the meanwhile, *miR-1* was found to bind with 3'UTR of *JARID2* mRNA to repress its expression, and JARID2 deficiency reduces PRC2 occupancy on cardiogenic genes. This conserved *miR-1*-JARID2 axis thus allows precise regulation of nuclear PRC2 occupancy on cardiogenic genes through *miR-1* activity in cytosol (Figure 3). In the cytoplasm, *HBL1* counteracts *miR-1*, which further determines mRNA and protein level of JARID2. After JARID2 protein entering nucleus, nuclear *HBL1* binds with JARID2 and EED to determine PRC2 occupancy on cardiogenic genes (Figure 3). Together, this *HBL1*/*miR-1-HBL1*/JARID2/PRC2 mechanism coordinates to fine-tune the chromatin state of essential cardiogenic genes in human cardiogenesis (8, 9).

*H19* has been well-studied in heart development and various heart diseases, including diabetic cardiomyopathy (12, 56), myocardial infarction (15), cardiac hypertrophy (6), muscular dystrophies (7) and heart failure (17). *H19* displays multiple functions in both cytoplasm and nucleus. Under cardiac hypertrophy, *H19* acts as a *miR-675* precursor to regulate the expression of miR-675 downstream gene VDAC1 and CM apoptosis in cytoplasm (Figure 3) (12); In nucleus, *H19* regulates PRC2 occupancy on the promoters of DIRAS3 and Tescalcin to repress cardiac hypotrophy (Figure 3) (6, 56). Consistently, all those studies reported that overexpression of *H19* in CMs can mitigate cardiac hypertrophy (6, 12, 56). These observations raise a question whether the cytosolic and nuclear functions of *H19* could coordinate to regulate cardiac hypertrophy, which remains to be further investigated.

*MDRL* (mitochondrial dynamic related lncRNA) is another well-studied lncRNA with defined mechanisms in both nucleus and cytoplasm. *MDRL* functions as a ceRNA of *miR-361*, which directly affects *miR-484* expression in mouse CMs (Figure 3) (48). *MDRL* inhibits mitochondrial fission and apoptosis through two miRNAs, *miR-361* and *miR-484*. In nucleus, *MDRL* affects the processing of *pre-miR-484* by targeting miR-361. In cytoplasm, *MDRL* regulates the mitochondrial network through both *miR-361* and *miR-484* (Figure 3). This work defined the complex functions of *MDRL* in both miRNA processing and downstream gene expression (48). All these findings suggest that clinical application of lncRNA should rely on deeper mechanistic studies, especially the differential roles of the same lncRNA in nucleus and cytoplasm.

## Conclusions

We summarized the biological functions and molecular mechanisms of lncRNAs in heart development and disease. In heart development, lncRNAs *Carmen, HBL1, Bvht, Fendrr, Bvht* and *CRP* regulate cardiac fate commitment, lineage differentiation, CM maturation/proliferation, and sarcomere organization etc. via both nuclear and cytoplasmic mechanisms. In heart diseases, lncRNAs are involved in the pathogenesis of cardiac hypotrophy, muscular dystrophy, myocardial infarction, diabetic cardiomyopathy, non-Ischemic cardiomyopathy (NICM) and heart failure and so on. With current progresses of genome-wide sequencing and functional screening studies, more functional lncRNAs have been identified in organogenesis and diseases, although the detailed molecular mechanisms of most lncRNAs have not been clearly defined. For example, lncRNAs *ALIEN* is expressed in undifferentiated pluripotent stem cells and impairs cardiovascular differentiation from pluripotent stem cells with molecular mechanism to be further studied (80). LncRNA *GASL1* is downregulated in chronic heart failure and can inhibit CM apoptosis through TGF-β1 signaling pathway, but how it regulates TGF-β1 is unclear (81). A group of lncRNAs are enriched in peripheral blood under different heart disease conditions (82, 83). For example, lncRNA *Heat2* expression is increased in the blood of heart failure patients (84); lncRNA *MT-LIPCAR*, transcribed from mitochondrial DNA, is positively associated with left ventricular diastolic dysfunction (54, 85). Although these lncRNAs might be utilized as disease markers or possess therapeutic penitential, their molecular mechanisms still require further characterizations.

The subcellular location of lncRNA is critical for its function, particularly for those lncRNAs highly expressed in both nucleus and cytoplasm (86). Cytosolic lncRNAs mainly function as regulators of mRNA stability, mRNA translation, miRNA processing and function, whereas nuclear lncRNAs can epigenetically regulate chromatin remodeling, structure, and gene transcription. Therefore, the balanced doses and transportation of lncRNA between cytoplasm and nucleus are expected to be a new research topic in the lncRNA field. During the last two decades, the translational potential of non-coding RNAs in heart disease therapy has gradually emerged. Nowadays, accumulated evidence indicates that lncRNAs provide a new layer of regulatory mechanism on top of coding genes. Since many lncRNAs have low evolutionary conservation (87), studies of lncRNAs might also reveal unique molecular mechanisms of heart development and disease in the human. Given the complex mechanisms, it is expected lncRNAs could offer new preventive and treatment approaches for human diseases including cardiovascular disease. Although, currently, there is no lncRNA therapeutic approach has progressed into preclinical or clinical trial, *H19* has been tested as a potential clinical therapeutic target in the Yucatan mini-pig (88). The expression changes of lncRNAs under different setting of heart diseases make it difficult for clinical applications. For example, in cardiac hypertrophy, *Mhrt* is downregulated (47), while *Chaer* and *Chrf* are upregulated (24, 38). *MALAT1* and *Whispr* expressions are upregulated in cardiac fibrosis, whereas *Meg3* and *GAS5* expressions are downregulated (89–92). Nevertheless, upregulated lncRNAs can be repressed by using shRNA, locked nucleic acids (LNAs) or GapmeR, and downregulated lncRNAs can be enhanced by using virus such as adenovirus, adeno-associated virus (AAV), and lentivirus (93). Although no clinical trial exists for lncRNA therapy in heart disease, the success of non-coding RNA *miR-132* based clinical trial paved the way. Recently, phase 1b clinical study to assess safety, pharmacokinetics and pharmacodynamics parameters of CDR132L, a *miR-132* inhibitor, has been completed (94). CDR132L is safe and well tolerated. Importantly, it improves cardiac function of heart failure patients. Therefore, the clinical applications of lncRNAs have a bright future, with fully and clearly characterized molecular mechanisms.

## Author Contributions

LH and LY summarized the references and prepared the manuscript. LH drafted the illustrations. LY supervised the project. All authors contributed to the article and approved the submitted version.

## Funding

This was supported by NIH (RO1 HL 147871, R21 HD095049) and AHA (20EIA35260114, 19TPA34850038) to LY, and 2019 AHA postdoc fellowship Award (19POST34380871) to LH.

## Conflict of Interest

The authors declare that the research was conducted in the absence of any commercial or financial relationships that could be construed as a potential conflict of interest.

## Publisher's Note

All claims expressed in this article are solely those of the authors and do not necessarily represent those of their affiliated organizations, or those of the publisher, the editors and the reviewers. Any product that may be evaluated in this article, or claim that may be made by its manufacturer, is not guaranteed or endorsed by the publisher.

## Abbreviations

lncRNA: long non-coding RNA
ceRNA: competing endogenous RNA
miRNAs: microRNAs
iPSCs: induced pluripotent stem cells
CPCs: cardiac precursor cells
CM: cardiomyocyte
CAD: coronary artery disease
NICM: non-Ischemic cardiomyopathy
PRC2: polycomb-repressive complex 2
AS: alternative splicing
JARID2: jumonji and AT-rich interaction domain containing 2
EED: embryonic ectoderm development
EZH2: enhancer of zeste homolog 2.
